# Social Rewards Enhance Offline Improvements in Motor Skill

**DOI:** 10.1371/journal.pone.0048174

**Published:** 2012-11-07

**Authors:** Sho K. Sugawara, Satoshi Tanaka, Shuntaro Okazaki, Katsumi Watanabe, Norihiro Sadato

**Affiliations:** 1 Division of Cerebral Integration, National Institute for Physiological Sciences, Okazaki, Aichi, Japan; 2 School of Life Sciences, The Graduate University for Advanced Studies (SOKENDAI), Hayama, Kanagawa, Japan; 3 Center for Fostering Young and Innovative Researchers, Nagoya Institute of Technology, Nagoya, Japan; 4 Research Center for Advanced Science and Technology, The University of Tokyo, Tokyo, Japan; 5 Japan Science and Technology Agency, CREST, Kawaguchi, Saitama, Japan; The Scripps Research Institute, United States of America

## Abstract

Motor skill memory is first encoded online in a fragile form during practice and then converted into a stable form by offline consolidation, which is the behavioral stage critical for successful learning. Praise, a social reward, is thought to boost motor skill learning by increasing motivation, which leads to increased practice. However, the effect of praise on consolidation is unknown. Here, we tested the hypothesis that praise following motor training directly facilitates skill consolidation. Forty-eight healthy participants were trained on a sequential finger-tapping task. Immediately after training, participants were divided into three groups according to whether they received praise for their own training performance, praise for another participant's performance, or no praise. Participants who received praise for their own performance showed a significantly higher rate of offline improvement relative to other participants when performing a surprise recall test of the learned sequence. On the other hand, the average performance of the novel sequence and randomly-ordered tapping did not differ between the three experimental groups. These results are the first to indicate that praise-related improvements in motor skill memory are not due to a feedback-incentive mechanism, but instead involve direct effects on the offline consolidation process.

## Introduction

Praise is the positive evaluation of another's products, performance, or attributes, where the evaluator presumes the validity of the standards on which the evaluation is based [1]. Praise can boost self-efficacy [2], [3], enhance feelings of competence and autonomy [4], create positive feelings [5], strengthen the association between responses and their positive outcomes [6], and provide incentives for task engagement [7]. In motor skill learning, for example, praise is hypothesized to provide feedback about the level of participant competence [8], which serves as an incentive to enhance practice efforts [9]. Thus, praise accelerates motor skill performance by enhancing motivation [8], [10], [11]. This is reasonable because motor skills are initially acquired by repeatedly performing an action during practice. However, learning a motor skill continues to evolve once practice ends [12], [13], [14] through consolidation, which is essential for skill formation and long-term retention [15], [16], [17]. There have been no investigations into the effects of praise on skill consolidation. Here, we hypothesize that praise influences the skill consolidation process directly, as opposed to indirectly through motivating further practice.

In the present study we tested this hypothesis through a behavioral experiment designed to manipulate both the timing of the praise given and the participants' expectation of a future test. First, to examine the effects of praise on offline rather than online performance improvements during training, participants were praised only after training was completed. Second, after a 24-h retention interval, all participants performed a “surprise” retest of the trained sequence. This minimized the possibility that the participants either physically or mentally practiced the trained sequence prior to the retest. These special considerations allowed us to investigate the direct benefits of praise on skill consolidation.

## Results

### Performance of the trained sequence

**Figure 1:**
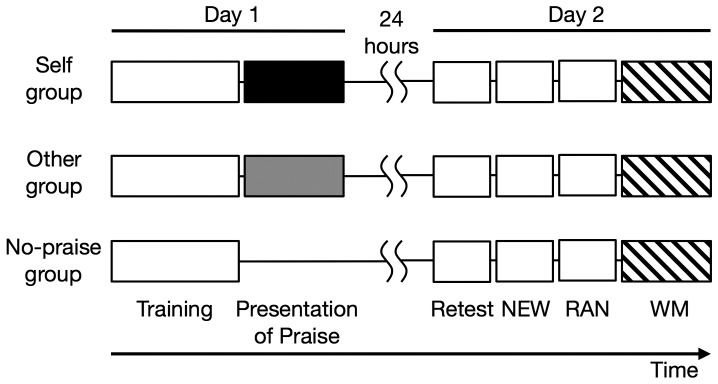
Experimental design. All participants were initially trained on a sequential finger-tapping task. They were then divided into three groups according to whether they received praise for their own training performance (Self group), praise for another participant's training performance (Other group), or no praise (No-praise group). The next day, participants completed a surprise retest of the trained sequence, a non-trained sequence (NEW), randomly-ordered tapping (RAN), and a working memory (WM) task.

**Figure 2:**
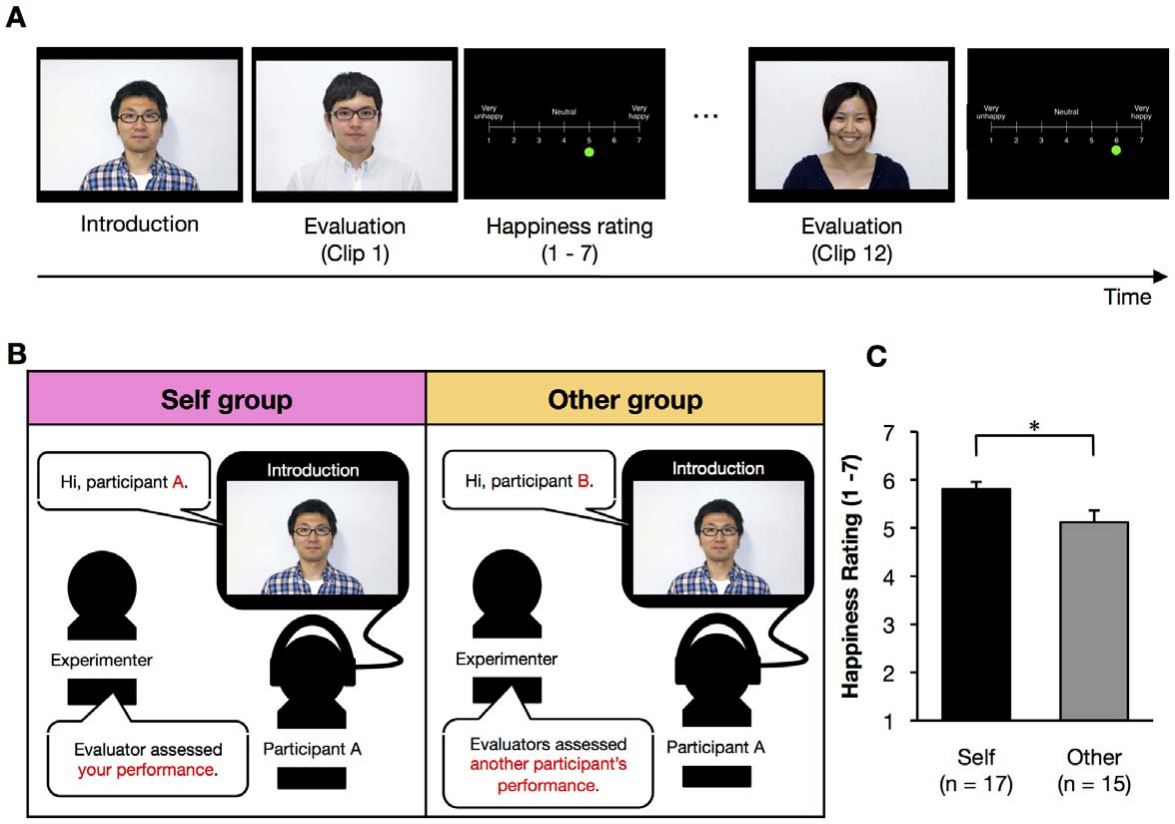
Manipulation of praise. (*A*) The sequence of events in the movie. The introduction clip was followed by 12 evaluation clips in a fixed order. After each evaluation clip, the participant was asked to rate their subjective happiness using a seven-point scale. (*B*) The instructions and the introduction clip differed between the Self and Other groups. In the Self group, participants were told that the movies represented an evaluation of their own training performance. Participants in the Other group were told that the movies represented the evaluation of another participant's performance. (*C*) The subjective judgment of participant happiness using a seven-point scale (1 = very unhappy, 4 = neutral, 7 = very happy). Error bars indicate the standard error of the mean (SEM). *p<0.05 (unpaired two-tailed t-test). The subject of the photograph has given written informed consent, as outlined in the PLoS consent form, to publication of their photograph.

Forty-eight right-handed participants came to the laboratory on two subsequent days (**Figure 1**). All participants were trained on a sequential finger-tapping task, for which offline improvement (a form of consolidation) has been described elsewhere [16]–[21]. Performance was defined as the number of correctly tapped sequences per 30-s trial. Immediately after training, in order to manipulate praise as an independent variable, participants were divided into three groups (**Figure 2**): in the “Self group” (*n* = 17), participants watched a movie in which the evaluators praised their own performance; in the “Other group” (*n* = 15), participants watched the same movie as the Self group, but were told that it represented the evaluation of another participant's performance; and in the “No-praise group” (*n* = 16), participants neither watched the movie nor received praise. Participant happiness after watching the clips was subjectively assessed using a seven-point scale (1 = very unhappy, 4 = neutral, 7 = very happy) and the ratings were significantly higher (happier) than 4 (the midpoint) in the Self group (black bar; one-sample t-test, *t*_16_ = 12.11, *p*<0.0001) and the Other group (gray bar; one-sample t-test, *t*_12_ = 4.58, *p*<0.001). To control out the positive word effect [22], we directly compared the happiness rate of both Self and Other groups. We were interested in the effect of the direction of the positive evaluation because when the positive evaluation is directed to “Self”, it should be perceived as praise, whereas it should not be when the positive evaluation is directed to “Other”. Indeed, participants in the Self group rated the movies as significantly more pleasant than those in the Other group (unpaired t-test, *t*_29_ = 2.50, *p*<0.05), indicating the successful manipulation of praise in present study.

**Figure 3:**
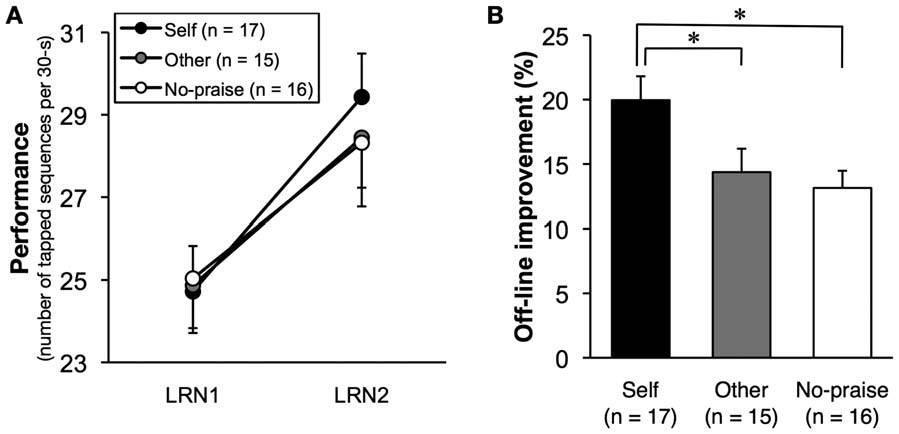
Results. (**A**) Mean performance during the last three training trials on day 1 (LRN1) and the first three retest trials on day 2 (LRN 2). All groups showed offline improvements on the trained sequence. (**B**) The rate of offline improvement, the percent increase from LRN1 to LRN2, was significantly greater in the Self group than the Other and No-praise groups. Black, gray, and white points or bars represent the Self, Other, and No-praise groups, respectively. Error bars indicate the standard error of the mean (SEM). *p<0.05 (Dunnett's test).

An analysis of variance (ANOVA) showed that performance at the end of training on day 1 did not significantly differ between the groups (*F*_2,45_ = 0.02, *p* = 0.98; **Figure 3** ***A***). In all groups, performance significantly improved between the end of training on day 1 and the retest on day 2 (*F*_1,45_ = 267.36, *p*<0.0001), confirming the offline improvement on the trained sequence (16, 17, 24–26). The rate of offline improvement differed significantly between the three groups (*F*_2,45_ = 3.53, *p*<0.05). Improvement was significantly greater in the Self group (19.95±1.85%; **Figure 3** ***B***) than in the Other group (14.37±1.33%, Dunnett's test, *p*<0.05) and the No-praise group (13.14±1.82%, *p*<0.05), indicating that praise enhanced skill consolidation.

Because several evidences showed that sex of the participants influence the consolidation and recall of different types of memory [23]–[25], it is possible that sex of participants interacted with the effect of praise in the offline performance improvements. Therefore, we conducted an additional ANOVA with Group (Self *vs* Other *vs* No-praise) and Sex (Male *vs* Female) as independent variables in the offline improvement. No significant main effect of Sex (*F_1,42_* = .05, *p* = .94) or interaction between Group and Sex (*F_2,42_* = .62, *p* = .52) was observed, while the effect of praise was significant (*F_2,42_* = 4.90, *p*<.05). Although present study was not designed to investigate the effect of sex differences, these results indicate that the effect of praise contributed to the offline improvements in motor skill independently of participants' sex.

In present study, we excluded a total of ten participants from the above-mentioned analyses because they suspected the movie (*n* = 5) or additionally practiced after the end of practice (*n* = 5). To evaluate the trend in the performance improvement of these excluded participants, we conducted an additional analysis of offline improvement rates in extra-experimental rehearsal group and suspicion group in comparison with that in the inclusion group (*n* = 48). According post-hoc test, relative to the average offline improvement rate of included participants (15.94±1.06%), that in extra-experimental rehearsal group was significantly higher (25.66±2.97%, *p*<.05, ANOVA with Dunnett's test) while that in participants who suspected for the movie did not significantly differ (16.96±2.55%, *p* = .94). These data suggest that extra-experimental rehearsal enhance the skill performance through additional exercise, and that suspicion for the movie *per se* did not influence the praise-related enhancement effect in skill consolidation.

### Performance on control tasks

An alternative explanation for the Self group's improvement was an increase in general motivation due to praise. To investigate this, the participants were asked to perform a non-trained sequence, a randomly-ordered tapping task, and a working memory task on day 2. There were no significant group differences in performance on either the non-trained sequence (Self, 22.12±0.92; Other, 21.98±1.03; No-praise, 23.27±0.97 sequences per trial; ANOVA: *F*_2,45_ = 0.52, *p* = 0.60) or the randomly-ordered tapping task (Self, 70.16±1.91; Other, 67.89±1.65; No-praise, 69.70±2.76 buttons per trial; *F*_2,45_ = 0.30, *p* = 0.74).

For the working memory task, there were no significant differences between the three groups in either reaction time (Self, 922±47 ms; Other, 912±35 ms; No-praise, 877±25 ms; *F*_2,33_ = 0.47, *p* = 0.63) or accuracy (the number of correct responses relative to all responses) (Self, 0.71±0.03; Other, 0.80±0.03; No-praise, 0.74±0.03; *F*_2,33_ = 1.77, *p* = 0.19).

### Sleep duration and quality during the night after training

Neither sleep duration (measured by subjective reports) nor actimetry measures differed between the groups (Subjective report, *F*_2,45_ = 0.02, *p* = 0.98; Actimetry, *F*_2,45_ = 0.52, *p* = 0.60). There were also no significant differences between the three groups in sleep quality, as calculated from physical activity during the night after training (Actimetry, *F*_2,45_ = 0.49, *p* = 0.62).

### Alertness, concentration, and fatigue during training and retest

Finally, there were no significant differences between the three groups for any of subjective ratings (sleepiness, concentration, and fatigue, ANOVA, *p* values ≥0.06), indicating that the differences in offline improvement between the groups were not caused by differences in subjective states during training or retest periods.

## Discussion

The purpose of this study was to investigate whether praise following motor training enhances skill consolidation. All groups showed offline skill improvements between the end of training and the retest 24 h later, confirming the results of previous studies [17], [19], [20]. Furthermore, our data indicated that praise following motor training enhances consolidation of the learned sequence since the rate of offline improvement was significantly greater in the Self group than in the Other or No-praise groups. As the evaluation video clips viewed by the Self and Other groups were identical except for the instructions indicating to whom the praise was directed, it is unlikely that any physical components in the video clips induced the observed group differences. In addition, other potential factors such as alertness, concentration, fatigue, and quality and duration of sleep did not differ between the groups, so cannot explain the improved consolidation in the Self group.

An alternative explanation of the present result is that praise induces a positive mood or increases the motivation to perform the motor task [5], [7], [10], resulting in the greater improvement in performance from day 1 to day 2 performance. If this were the case, however, it would be expected that the uneven performance between the three groups would occur not only for the trained sequence but also on the other tasks. However, the present results showed no significant group differences in these tasks, suggesting that the effects of praise following training were specific to the trained sequence rather than a more general effect on experimental task performance.

Praise is regarded as a reward [26], because praise has two essential components of reward, that is, hedonic and motivational [27]. Praise can induce a feeling of happiness (hedonic component), and also promotes motivation (motivational component) [8], [10], [11]. A recent human neuroimaging study demonstrated that praise activates reward-related areas of the brain, specifically the ventral striatum [26]. Rewards are associated with increased dopaminergic activity in the midbrain and striatum, in which dopamine-dependent long-term potentiation (LTP) [28]–[30] has an important role in memory consolidation. The cortico-striatal system plays a critical role in the automatization of the type of motor sequence learning used in the present study [18], [31], [32]. Synaptic plasticity represented by LTP at cortico-striatal synapses strongly depends on the activation of dopamine circuits [33]. As the ventral striatum is the part of the reward system driven by dopamine [34], rewards are expected to affect motor skill consolidation. Taken together, present findings suggest that praise functions as “social reward” that induces the dopamine transmission in the striatum, resulting in an enhancement of the motor skill consolidation.

Sleep is another possible contributing factor. There is mounting evidence that sleep is necessary for the offline improvement in the sequential finger-tapping task used in the present investigation [16]–[21]. Although this study was not designed to determine whether sleep is necessary for the praise-related enhancement of skill consolidation, it is reasonable to expect that this enhancement selectively occurs during sleep. Consolidation of a new motor sequence during sleep appears to rely on the covert re-activation of the brain regions that were initially involved in learning the motor skill [35]. Recent human neuroimaging studies have shown that several brain areas that were activated during the execution of a memory task are significantly re-activated during sleep [35]–[37], and that such re-activation facilitates memory consolidation [35], [36]. Furthermore, a previous animal study revealed that sleep-dependent re-activation of firing patterns in the ventral striatum took place after reward-related learning [38]. In line with these findings, it is conceivable that the cortico-striatal loop that is modified by praise after the training is then re-activated during sleep, which in turn contributes to the praise-related enhancement of offline, overnight consolidation. This working hypothesis will be the focus of future experimental investigations.

In summary, the present study demonstrated that social rewards directly enhance skill consolidation in humans, and suggests that they have a novel functional effect on the human motor memory system. Further understanding of the effects of social rewards on skill consolidation could help to develop protocols to improve motor skills in educational and rehabilitative contexts.

## Materials and Methods

### Participants

Written informed consent was obtained from all participants before participation in the experiment and the study conducted according to the Declaration of Helsinki. If participant was a minor (i.e., 18 or 19 year-old), two different experimenters ensured their ability to make decision and obtained their written informed consent to the participation of this experiment, which were approved by the internal review board of Research Center for Advanced Science and Technology, The University of Tokyo. Fifty-eight healthy volunteers (39 male and 19 females, mean [M] ± standard deviation [SD] = 22.6±4.67 years) participated in this study. None of the participants had a history of neurological, psychiatric, or sleep disorders, and none had had previous training in playing the piano. Based on interviews after the experiments, five participants were excluded from the analyses because they physically or mentally practiced the trained motor sequence after the end of training on day 1. Another five participants were excluded because they noticed or suspected that the evaluation movies that they watched were predetermined. Thus, data from 48 participants (35 males and 13 females; M ± SD = 22.8±5.17 years) were used for analysis (Self group, *n* = 17; Other group, *n* = 15; No-praise group, *n* = 16).

### Experimental Procedure

Participants came to the laboratory on two subsequent days. All participants trained on a sequential finger-tapping task [12], [18]–[21], [39], [40] on day 1. The participants were told that evaluators in another room were monitoring their performance through a web camera above the computer monitor, and would comment on their performance after training. However, in reality, their performance was not monitored. After training, all participants received visual feedback about their performance (for example, their learning curve). The participants were then divided into three groups to systematically manipulate the praise that they experienced: 1) participants who watched a movie in which evaluators praised their training performance (Self group); 2) participants who watched the same movie as the Self group, but who were told that it reflected the evaluation of another participant's performance (Other group); and 3) participants who did not watch the movie and who received no praise (No-praise group).

Unbeknownst to the participants, the contents of the movie were predetermined and prerecorded, with actors and actresses portraying the evaluators. At the end of the experiment on day 1, participants were told that they would perform a different task on the next day. On the following day, however, all participants performed a “surprise” retest of the trained sequence; this was intended to minimize the possibility that the participants either physically or mentally practiced the trained sequence prior to the retest, or that those in the Self group, in particular, were more motivated to perform the tasks on day 2. We then examined the effect of the manipulation of praise on the retest performance of the trained sequence.

After the retest, the participants also performed a non-trained sequence, a randomly-ordered tapping task and completed a working memory task. These additional tasks were included to investigate whether the effects of praise were specific to the offline improvement in the trained sequence or induced a more general feeling of happiness that increased motivation to perform well on day 2. If praise enhanced general motivation in the Self group, performance on all additional tasks on day 2 should be better in the Self group than in the Other and No-praise groups.

### Sequential Finger Tapping Task

The sequential finger tapping task required participants to press four numeric keys on a standard computer keyboard repeatedly with the fingers of their non-dominant (left) hand as quickly and as accurately as possible for 30-s periods (for details, see [19], [21]). On day 1, one-half of the participants trained on sequence A (“4-1-3-2-4”), whereas the others trained on sequence B (“2-3-1-4-2”). Training on day 1 consisted of 12 30-s trials with 30-s rest periods between trials, whereas the retest on day 2 consisted of five trials with the same rest interval.

Finger tapping performance was evaluated by the number of correctly tapped sequences per 30-s trial. The offline performance improvement following a night of sleep was defined as the percent increase in mean performance from the last three trials during training on day 1 compared with the first three retest trials on day 2 [18]–[20], [39], [40].

On day 2, participants also performed the sequence that they had not received training on during day 1 (that is, a participant who trained on sequence A on day 1 performed sequence B on day 2), and the randomly-ordered tapping task, in which stimuli were presented in a random order. Both tasks consisted of five 30-s trials with a 30-s rest period between trials. Performance for the non-trained sequence (NEW) and the randomly-ordered (RAN) tapping was calculated based on the mean number of correctly tapped sequences (NEW) or correctly pressed buttons (RAN) during the five trials.

### Manipulation of Praise

After the training on day 1, participants in the Self and Other groups watched a movie in which evaluators praised the training performance. We adopted a movie instead of live praise because predetermined movie can totally control out the variability of evaluators' comments and non-verbal information such as facial expression and intonation. Participants in the Self group were told that the movie represented the evaluation of their own performance during training. The movie consisted of three components: one introduction clip, 12 evaluation clips, and happiness ratings for each clip. In the introduction clip, a man greeted the participant by name to make the evaluation appear more believable and meaningful. Each movie clip was pre-recorded using six actors and six actresses. Ten movie clips contained positive feedback, and two neutral movie clips were included to maintain the attention of participants by making the evaluation less predictable.

In the evaluation movies, praise was directed at the participant's training performance, their attitude during training, or their social ranking relative to other participants (see **Table 1** for examples of evaluators' comments used in this experiment). To rule out the possibility that simply watching the movie might influence the offline improvement in motor skill, we included the Other group, in which participants watched the same movie clips but were told that they represented the evaluation of another participant's training performance. In the introduction clip seen by the Other group, a man used another participant's name. In both the Self and Other groups, regardless of the target of praise, the participants were asked to rate how happy they felt upon watching each movie clip using a seven-point scale (1 = very unhappy, 4 = neutral, and 7 = very happy; the responses for one participant were not collected due to technical difficulties). The order of the evaluation clips was fixed across participants.

**Table 1:** Examples of the comments from evaluation clips.

Valence of comment	Direction of evaluation	Content
Positive	Performance, Social Ranking	Hi, I observed your performance and attitude during the motor task. The tapping became more rhythmical over time. Your performance was great. The number of the pressed buttons in the last trial might be the highest of all the participants I have observed. Thank you.
Positive	Attitude, Social Ranking	Hello. I would like to comment on your wonderful performance. First, I think your motor performance on the last trial was the highest of all the participants. In addition, you concentrated very well during the motor task. Thanks for your participation.
Positive	Performance, Social Ranking	I can imagine that other evaluators will also give you good feedback. Actually, your performance was amazing and deserves praise. Among the previous participants in the experiment, your performance was the best. Thank you.
Neutral	Performance, Social Ranking	Thanks for your participation. The total number of tapped buttons and the speed of tapping increased as practice progressed. Your performance was average relative to the other participants.

After the experiment on day 2, the participants were interviewed to determine whether they had any doubts about the evaluation movies they watched. After this, all participants were fully debriefed.

### Working Memory Task

A subset of the participants (*n* = 35) performed an object working memory task on day 2. A previous study indicated that performance on working memory tasks is highly sensitive to a participant's motivational state [41]. In the delayed-matching working memory task, participants were asked to remember three irregular polygons, and were then required to decide while whether a probe stimulus matched any of the three target stimuli (for details, see [41]). The task was presented in a total of 84 trials.

### Alertness, Concentration, and Fatigue During Training and Retest

As it was possible that the subjective state of the participants during training and retest might influence their performance, they completed questionnaires to rate their level of alertness (Stanford Sleepiness Scale rating, [42], translated into Japanese), concentration (1 = not at all, 7 = very concentrated), and fatigue (1 = high level of fatigue, 7 = no fatigue, [43]) using a seven-point scale at the end of the training and retest periods.

### Sleep Duration and Quality the Nights Before and After Training

Because sleep plays an important role in the offline improvement of motor skills [16], [18]–[21], sleep duration the night after training was measured by subjective reports and actimetry. Participants were also asked to report the time that they went to bed both the night before and after training, and the time that they woke up on the training and retest mornings. In addition, to confirm the validity of the subjective sleep-duration reports, the physical activity of a subset of participants (*n* = 26, due to the limited number of available actimetry sensors) was measured from the end of training to the retest time using a standard actimetry sensor. There was a significant correlation between the duration of sleep reported by the participant and that measured by actimetry (Pearson's correlation, *r*_26_ = 0.81, *p*<0.0001), confirming that the duration of sleep calculated from the subjective reports was reliable. We defined sleep quality as the percentage of true sleep epochs relative to the total sleep intervals automatically determined by AW2 software (Ambulatory Monitoring, Inc., New York).

### Statistical Analysis

Statistical analyses were based on a general linear model using analyses of variance (ANOVAs) for independent or repeated measures. Dunnett's test (two-tailed; compared with the Self group) was adopted for multiple-planned comparisons [44], [45], based on the hypothesis that the offline improvement in motor skill in the Self group was significantly greater than in the Other and No-praise groups. Analysis of happiness ratings was performed using unpaired t-tests (two-tailed). All analyses were performed using SPSS 19.0 software and the level of significance was *p*<0.05.
